# Expression of oxidized protein tyrosine phosphatase and γH2AX predicts poor survival of gastric carcinoma patients

**DOI:** 10.1186/s12885-018-4752-4

**Published:** 2018-08-20

**Authors:** Usama Khamis Hussein, Ho Sung Park, Jun Sang Bae, Kyoung Min Kim, Yun Jo Chong, Chan Young Kim, Keun Sang Kwon, Myoung Ja Chung, Ho Lee, Myoung Jae Kang, Woo Sung Moon, Kyu Yun Jang

**Affiliations:** 1 Department of Pathology, Chonbuk National University Medical School, Research Institute of Clinical Medicine of Chonbuk, National University-Biomedical Research Institute of Chonbuk National University Hospital, Jeonju, Republic of Korea; 2 Research Institute for Endocrine Sciences, Chonbuk National University Medical School, Jeonju, Republic of Korea; 3 Faculty of Science, Beni-Suef University, Beni-Suef, Egypt; 4 Center for University-wide Research Facilities, Chonbuk National University, Jeonju, Republic of Korea; 5 Department of Surgery, Chonbuk National University Medical School, Jeonju, Republic of Korea; 6 Department of Preventive Medicine, Chonbuk National University Medical School, Jeonju, Republic of Korea; 7 Department of Forensic Medicine, Chonbuk National University Medical School, Jeonju, Republic of Korea

**Keywords:** Stomach, Carcinoma, Oxidative stress, Protein tyrosine phosphatase, γH2AX

## Abstract

**Background:**

Oxidative stress induces various intracellular damage, which might be correlated with tumorigenesis. Accumulated oxidative stresses might inactivate protein tyrosine phosphatase (PTP) by oxidizing it, and inducing the phosphorylation of H2AX (γH2AX) in response to DNA damage.

**Methods:**

We evaluated the clinical significance of the expression of oxidized-PTP and γH2AX in 169 gastric carcinomas.

**Results:**

Immunohistochemical expression of nuclear oxidized-PTP, cytoplasmic oxidized-PTP, and γH2AX expression were significantly associated with each other, and their expressions predicted shorter survival of gastric carcinoma patients. In multivariate analysis, nuclear oxidized-PTP (overall survival; *p* <  0.001, relapse-free survival; *P* <  0.001) was an independent indicator of poor prognosis of gastric carcinoma patients. In addition, co-expression patterns of nuclear oxidized-PTP and γH2AX were independent indicators of poor prognosis of gastric carcinoma patients (overall survival; *P* <  0.001, relapse-free survival; *P* <  0.001).

**Conclusions:**

This study suggests that oxidative stress-mediated oxidation of PTP might be involved in the progression of gastric carcinomas. In addition, this study suggests that individual and co-expression pattern of nuclear oxidized-PTP and γH2AX might be used as a prognostic marker of gastric carcinomas.

## Background

Protein tyrosine phosphatases (PTPs) control phosphorylation of tyrosine residues and are subdivided by their localization as either transmembrane receptor-like PTPs or non-transmembrane receptor-like PTPs [1, 2]. By regulating the phosphorylation status of various proteins, PTPs are involved in various cellular processes such as cellular proliferation, differentiation, apoptosis, adhesion, motility, and tumorigenesis [2, 3]. Especially, their role in antagonizing tyrosine kinases makes them of interest for their role in tumorigenesis [3]. With regards to the regulation of phosphorylation of proteins involved in various cellular processes, the balance of phosphatase activity and kinase activity might be important in the tumor microenvironment [3]. For the treatment of human cancers, regulation and/or inhibition of oncogenic tyrosine kinases have been extensively investigated and development of specific inhibitors of kinases is a rapidly growing field in new drug development [4]. Therefore, the activity of PTPs might be an important target in the regulation of cancers. Usually, the activity of PTP can be regulated by several mechanisms such as oxidation, proteolysis, phosphorylation, and sumoylation [2]. Among these, oxidative stress-mediated oxidation of PTPs is one of the important mechanisms silencing phosphatase activity of PTPs [1, 5]. However, with transient oxidative stress, the oxidation of PTPs is reversible and decreased by reducing agents [1, 2, 6]. However, continuous exposure to reactive oxygen species (ROS) induces hyper-oxidation of PTPs to form biologically irreversible sulfinic and sulfonic acid states in PTPs [1, 2, 6]. Therefore, detection of oxidized-PTP (Ox-PTP) might be indicative of consecutive exposure to oxidative stresses [6]. Moreover, oxidation of PTPs is common in human cancers and influences tumorigenesis as seen in PTEN inactivation by oxidation [3, 7, 8]. In addition, oncogenic mutation induces ROS production and that enhances mitogenic and mutogenic signaling to contribute to tumor development [9–11].

Another aspect of the biologic effects of oxidative stresses is that they are genotoxic and induce DNA damage. When there is DNA damage, various pathways are activatyed which determine whether apoptosis or repair of damages will be activated [12–14]. Moreover, accumulated DNA damage could induce genomic instability and eventually various mutations could initiate tumorigenesis [15, 16]. Under these conditions, various metabolic antioxidant defenses and a sophisticated system of DNA damage response (DDR) are activated [17, 18]. When DNA damage repair responses operate, phosphorylation of H2AX is one of the sensitive indicators of DNA repair [12–14]. Therefore, estimation of phosphorylated-H2AX (γH2AX) expressing foci might be an indicator of DNA damage under oxidative stress [19]. In addition, the expression of DDR molecules including γH2AX was associated with poor prognosis of various type of human cancers [20–23]. This might be associated with the fact that DDR molecules contribute to resistance to genotoxic anti-cancer therapies and the expression of γH2AX has been proposed as a useful prognostic marker for cancer patients [19, 21, 22].

Gastric cancer is one of the most common and deadliest malignancies worldwide and represents the second cause of cancer-related death [24, 25]. Based on the tumor suppressive role of PTP, the expression of PTPs has been investigated in gastrointestinal disease, and dysfunction of PTPs has been observed in gastric cancer [26, 27]. In contrast, the expression of PRL-3 was increased in metastatic lesions of gastric cancer, which suggests PRL-3 as a tumor promotor [28]. However, there have been limited studies on the expression of PTPs and DDR molecules in gastric cancers. In addition, there no reports have focused on the detection of Ox-PTP as a prognostic marker of human malignant tumors. When considering the common involvement of ROS in both PTP oxidation and DNA damage, evaluation of the expression of Ox-PTP and γH2AX might be indicative for evaluation of cells under oxidative stress-mediated cellular damage. Based in this rationale, we investigated the expression of Ox-PTP and γH2AX in human gastric carcinoma tissue samples and evaluated the utility of the expression of Ox-PTP and γH2AX as prognostic markers of gastric carcinomas.

## Methods

### Gastric carcinoma patients and tissue samples

In this study, we evaluated one hundred and sixty-nine cases of gastric carcinomas from the177 cases that we have used in our previous study. Originally, 177 cases of gastric carcinoma were selected from 200 cases derived by matching stage I, II, and III patients to 50 cases of stage IV with calendar year of surgery (±2 y), gender, and age (±2 y) [29]. Cases were matched according to the 6th edition of the American Joint Committee Cancer Staging System [30]. Thereafter, eight cases were missing available tissue microarray cores. In these eight cases, there were no available original tissue block. Therefore, 169 gastric carcinoma patients who performed therapeutic radical gastrectomy between January 1997 and December 2005 in Chonbuk National University Hospital were included in this study. All cases were classified and staged according to WHO classification [24] and tumor stage was re-evaluated by the 8th edition of American Joint Committee Cancer Staging System [31]. In sum, 34 cases of stage I, 44 cases of stage II, 64 cases of stage III, and 27 cases of stage IV gastric carcinomas were evaluated in this study. The clinicopathological information of the patients was gathered and reviewed from the routine medical records and histopathologic reports. According to the classification patterns of the WHO, the patients’ clinical information was grouped based on sex, age, blood serum carcinoembryonic antigen (CEA) and cancer antigen 19–9 (CA 19–9), histologic tumor grade, lymph node metastasis, distance metastasis, and venous invasion. Tumor invasion and Lauren classification criteria were based on early versus advanced gastric carcinoma.

### Gastric cancer cells and western blot

To validate anti-Ox-PTP and anti-γH2AX antibodies, we performed western blotting analysis using the NCI-N87, MKN45, and KATO-III (Korean Cell Line Bank, Seoul, Korea) human gastric cancer cell line. NCI-N87 and MKN-45 cells were cultured in RPMI1640 media, and KATO-III cells were cultured in IMDM media. To induce oxidative stress, cells were treated with 400 μM H_2_O_2_ for 24 h. The cells were lysed with PRO-PREP Protein Extraction Solution buffer (iNtRON Biotechnology Inc., Seongnam, Korea) with 1% protease and phosphatase cocktails inhibitor 2,3 (Sigma-Aldrich, St. Louis, MO, USA). The total protein was probed with primary antibodies for Ox-PTP active site (Research and Diagnostic systems Inc., Minnesota, MN), γH2AX (Ser 139) (Cell Signaling Technology, Beverly, MA), and actin (Sigma, St. Louis, MO).

### Immunohistochemical staining and scoring

Immunohistochemical staining was performed on tissue microarray sections with one 3.0 mm core for each case. Antigen retrieval was performed by boiling tissue microarray sections with microwave oven in pH 6.0 antigen retrieval solution (DAKO, Glostrup, Denmark) for 20 min. The primary antibodies for the Ox-PTP active site (1:100, Research and Diagnostic systems Inc., Minnesota, MN) and γH2AX (Ser 139) (1:100, Cell Signaling Technology, Beverly, MA) were used for immunostaining. Immunohistochemical staining was scored by two pathologists (KYJ and HSP) with consensus under a multi-viewing microscopy without knowledge of the clinicopathological information. Immunohistochemical staining for Ox-PTP was scored according to Allred scoring by adding staining intensity (0; no, 1; weak, 2; intermediate, 3; strong) and area (0; 0%, 1; 1%, 2; 2–10%, 3; 11–33%, 4; 34–66%, 5; 67–100%) [21, 32, 33]. The score for Ox-PTP staining ranged from zero to eight. Immunohistochemical staining for γH2AX was scored by counting the number of cells stained for γH2AX in five high-power fields (magnification; × 400, area of one high-power field; 0.238 mm^2^) [19, 21, 23].

### Statistical analysis

The cut-off points for the immunohistochemical staining score were determined by receiver operating characteristic curve analysis. The point with the highest area under the curve to predict overall survival (OS) of gastric carcinoma patients was determined as a cut-off point. Survival analysis was evaluated for the OS and relapse-free survival (RFS). The follow-up end point of this study was December 2012. In OS analysis, death of the patients from gastric carcinoma was event in analysis, and the patients who were alive at last follow-up were treated as censored. Relapse of tumor or death from gastric carcinoma was an event in RFS analysis, and the patients who alive without relapse at last follow-up date were treated as censored. Univariate and multivariate Cox proportional hazards regression, Kaplan-Meier survival analysis, and Pearson’s chi-square test were performed using SPSS software (IBM, version 20.0, CA). *p* values less than 0.05 were considered statistically significant.

## Results

### The expression ox-PTP and γH2AX in human gastric carcinoma and their association with clinicopathological characteristics

**Fig. 1:**
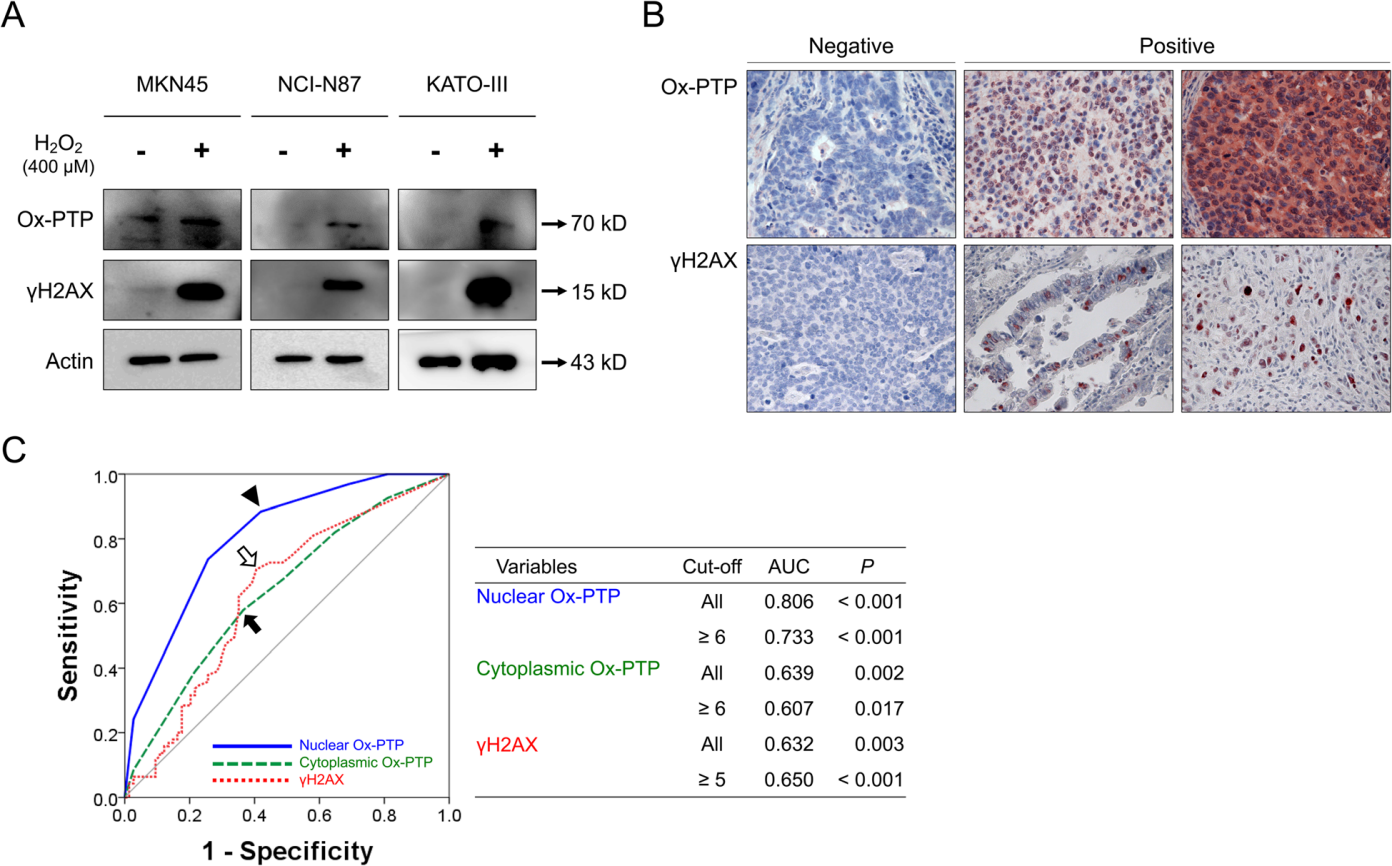
The expression of Ox-PTP and γH2AX in human gastric carcinomas, and statistical analysis. **a** Western blotting for Ox-PTP and γH2AX to validate antibodies. The expression of Ox-PTP and γH2AX increased with treatment of 400 μM H_2_O_2_ for 24 h in NCI-N87, MKN45, and KATO-III gastric cancer cells. **b** Ox-PTP is expressed in both the cytoplasm and the nuclei of tumor cells, and the expression of γH2AX is observed in the nuclei of tumor cells. Original magnification, × 400. **c** Receiver operating characteristic curve analysis for the estimation of cut-off points for the staining scores of Ox-PTP and γH2AX. The highest positive cut-off points for the nuclear expression of Ox-PTP (arrow head), cytoplasmic expression of Ox-PTP (black arrow), and γH2AX (empty arrow) were estimated at the highest area under the curve (AUC) value for the estimation of death and survival events of patients. The likelihood points for the immunohistochemical staining score of nuclear Ox-PTP, cytoplasmic Ox-PTP, and γH2AX expression were 6, 6, and 5, respectively

To validate antibodies for Ox-PTP and γH2AX, western blotting was performed in MKN45 gastric cancer cells after inducing oxidative stress with 400 μM H_2_O_2_. The H_2_O_2_-induced oxidative stress increased the expression of Ox-PTP and γH2AX in MKN45 cells (Fig. 1a). Immunohistochemically, the expression of Ox-PTP was seen in both the nuclei and the cytoplasm of the tumor cells. The expression of Ox-PTP was classified as nuclear-dominant, cytoplasmic dominant, or expression in both nuclei and cytoplasm (Fig. 1b). Therefore, the expression of Ox-PTP was separately scored according to its nuclear expression (Nu-Ox-PTP) and cytoplasmic expression (Cy-Ox-PTP). In contrast, the expression of γH2AX was exclusively expressed in the nuclei of tumor cells and the number of γH2AX-positive cells ranged from zero to 560 (median; 7, mean; 32, standard deviation; 77). The cut-off points of the immunohistochemical scores for the Nu-Ox-PTP, Cy-Ox-PTP, and γH2AX expression were 6, 6, and 5 (Fig. 1c). In these cut-off values, 68% (115/169), 49% (82/169), and 57% (97/169) of cases were categorized as Nu-Ox-PTP-positive, Cy-Ox-PTP-positive, and γH2AX-positive groups, respectively (Table 1). Regarding Ox-PTP positivity, it was shown that nuclear expression was significantly associated with more clinical variables than cytoplasmic expression. Nu-Ox-PTP positivity was significantly associated with TNM stage (*P* <  0.001), T category of stage (*p* = 0.012), N category of stage (*P* = 0.001), tumor invasion (*P* = 0.003), and histological grade (*P* = 0.002). Moreover, a strong positive correlation was observed between Nu-Ox-PTP positivity and both Cy-Ox-PTP positivity (*P* <  0.001) and γH2AX positivity (*P* <  0.001) (Table 1). Cy-Ox-PTP positivity was significantly associated with WHO classification (*P* = 0.030), Lauren classification (*P* <  0.001), and γH2AX positivity (*P* = 0.031). γH2AX positivity was significantly associated with age of patients (*P* <  0.001), pre-operative carcinoembryonic antigen level (*P* = 0.022), T category of stage (*P* = 0.043), and Lauren classification (*P* = 0.037).

**Table 1:** Clinicopathologic variables and the expression of nuclear and cytoplasmic expression of expression of Ox-PTP and γH2AX in gastric carcinoma patients

Characteristics		No.	Ox-PTP, nucleus		Ox-PTP, cytoplasm		ɣH2AX	
			Positive	*P*	Positive	*P*	Positive	*P*
Age (years)	< 60 y	52	34 (65%)	0.621	27 (52%)	0.555	18 (35%)	< 0.001
	≥ 60 y	117	81 (69%)		55 (47%)		79 (68%)	
Sex	Female	41	28 (68%)	0.969	16 (39%)	0.162	23 (56%)	0.847
	Male	128	87 (68%)		66 (52%)		74 (58%)	
CEA^a^	Normal	110	74 (67%)	0.463	50 (45%)	0.215	60 (55%)	0.022
	Elevated	31	23 (74%)		18 (58%)		24 (77%)	
CA19–9^a^	Normal	124	85 (69%)	0.599	62 (50%)	0.346	72 (58%)	0.193
	Elevated	16	12 (75%)		6 (38%)		12 (75%)	
T category	1	36	17 (47%)	0.012	16 (44%)	0.555	17 (47%)	0.550
	2	17	10 (59%)		7 (41%)		10 (59%)	
	3	20	15 (75%)		13 (65%)		11 (55%)	
	4a	73	53 (73%)		34 (47%)		43 (59%)	
	4b	23	20 (87%)		12 (52%)		16 (70%)	
N category	0	60	29 (48%)	< 0.001	26 (43%)	0.074	28 (47%)	0.043
	1	22	16 (73%)		8 (36%)		11 (50%)	
	2	33	21 (64%)		14 (42%)		19 (58%)	
	3	54	49 (61%)		34 (63%)		39 (72%)	
M category	0	165	112 (68%)	0.763	80 (48%)	0.952	94 (57%)	0.471
	1	4	3 (75%)		2 (50%)		3 (75%)	
TNM stage	I	34	15 (44%)	< 0.001	14 (41%)	0.777	15 (44%)	0.188
	II	44	25 (57%)		21 (48%)		24 (55%)	
	II	64	52 (81%)		33 (52%)		39 (61%)	
	IV	27	23 (85%)		14 (52%)		19 (70%)	
Venous invasion	Absence	139	92 (66%)	0.264	66 (47%)	0.561	76 (55%)	0.124
	Presence	30	23 (77%)		16 (53%)		21 (70%)	
WHO classification	Tubular	115	86 (75%)	0.091	66 (57%)	0.030	72 (63%)	0.055
	SRC	18	11 (61%)		6 (33%)		9 (50%)	
	Mucinous	17	7 (41%)		4 (24%)		5 (29%)	
	Mixed	15	9 (60%)		4 (27%)		7 (47%)	
	Papillary	2	1 (50%)		1 (50%)		2 (100%)	
	Neuroendocrine	2	1 (50%)		1 (50%)		2 (100%)	
Histologic grade^b^	WD	10	4 (40%)	0.002	7 (70%)	0.182	7 (70%)	0.248
	MD	64	55 (86%)		40 (63%)		44 (69%)	
	PD	43	28 (65%)		20 (47%)		23 (53%)	
Lauren classification	Intestinal	71	54 (76%)	0.079	47 (66%)	< 0.001	48 (68%)	0.037
	Diffuse	73	43 (59%)		25 (34%)		34 (47%)	
	Mixed	25	18 (72%)		10 (40%)		15 (60%)	
ɣH2AX	Negative	72	38 (53%)	< 0.001	28 (39%)	0.031		
	Positive	97	77 (79%)		54 (56%)			
Ox-PTP, cytoplasm	Negative	87	38 (44%)	< 0.001				
	Positive	82	77 (94%)					

Abbreviations: *CEA* carcinoembryonic antigen, *CA19–9* carbohydrate antigen 19–9, *LN* lymph node, *WD* well differentiated, *MD* moderately differentiated, *PD* poorly differentiated, *SRC* signet ring cell carcinoma, *EGC* early gastric cancer, *AGC* advanced gastric cancer; ^a^Preoperative serum level of CEA or CA19–9 were not measured in 28 and 29 patients, respectively. ^b^Histologic grading was carried in tubular and papillary type carcinomas according to the grading system of the WHO histological classification of gastric tumours

### The expression of ox-PTP and γH2AX were associated with shorter survival of gastric carcinoma patients

**Fig. 2:**
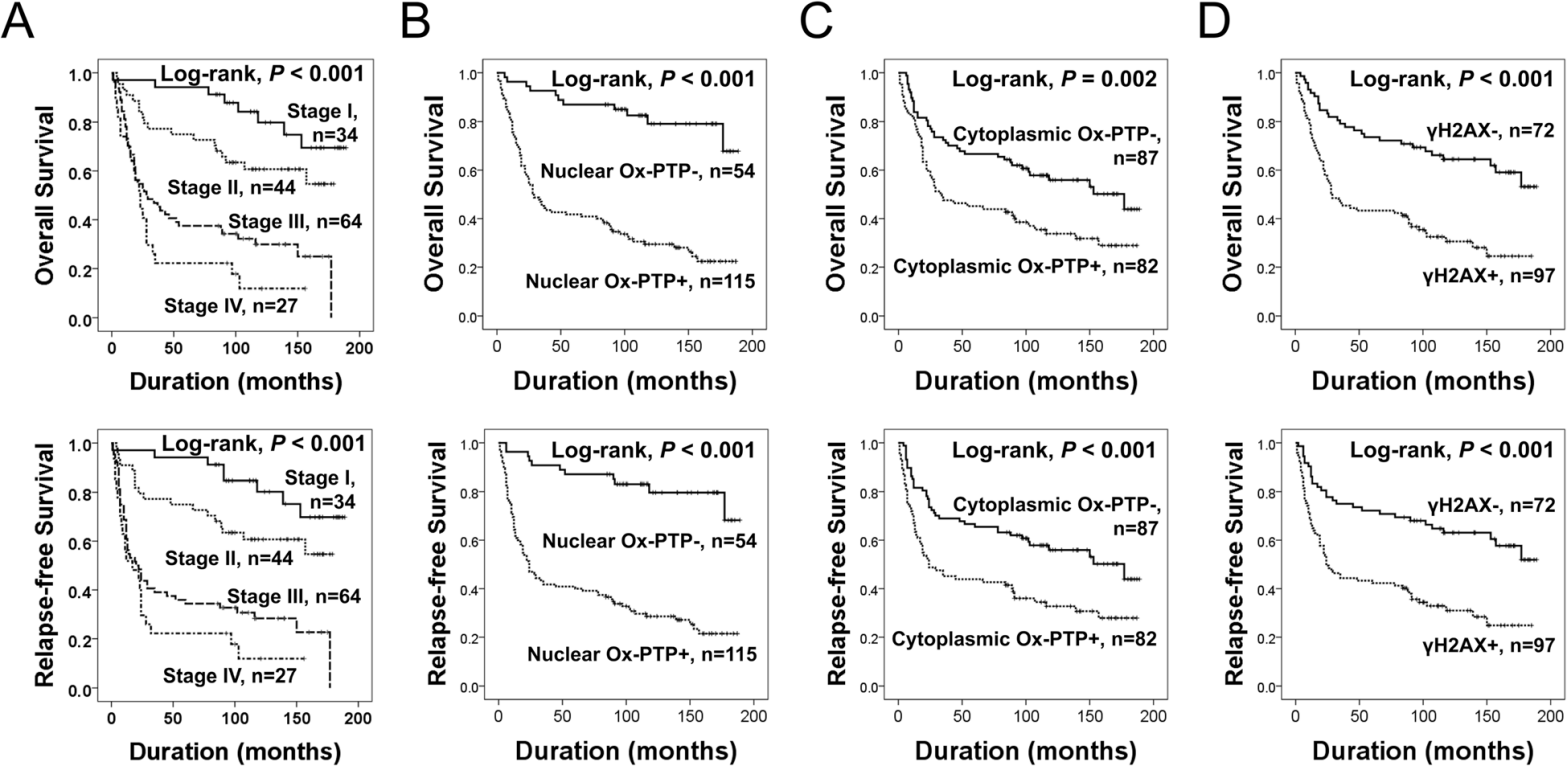
Kaplan-Meier survival analysis in gastric carcinomas. Overall survival and relapse-free survival in 169 patients according to the TNM stage (**a**), nuclear expression of Ox-PTP (**b**), cytoplasmic expression of Ox-PTP (**c**), and the expression of γH2AX (**d**)

The clinicopathological factors that showed significant associations with shorter OS and RFS using univariate analysis of gastric carcinoma patients were elevations of serum levels of CEA (OS; *P* = 0.019, RFS; *P* = 0.021) and CA19–9 (OS; *P* = 0.022, RFS; *P* = 0.034), advanced tumor stage (OS; overall *P* <  0.001, RFS; overall *P* <  0.001), venous invasion (OS; *P* <  0.001, RFS; *P* <  0.001), advanced gastric cancer type (OS; *P* <  0.001, RFS; *P* <  0.001), Nu-Ox-PTP positivity (OS; *P* <  0.001, RFS; *P* <  0.001), Cy-Ox-PTP positivity (OS; *P* = 0.003, RFS; *P* <  0.001), and positivity of γH2AX (OS; *P* <  0.001, RFS; *P* <  0.001) (Table 2). The greatest risk for shorter OS and RFS was seen in patients who had tumors positive for nuclear expression of Ox-PTP. Nu-Ox-PTP positivity predicted a 5.799-fold [95% confidence interval (95% CI): 3.084–10.905, *P* <  0.001] greater risk of death and a 5.954-fold (95% CI: 3.167–11.192, *P* <  0.001) greater risk of death or relapse. Cy-Ox-PTP positivity also predicted a 1.863-fold (95% CI: 1.238–2.803, *P* = 0.003) greater risk of shorter OS and a 1.982-fold (95% CI: 1.319–2.978, *P* <  0.001) greater risk of shorter RFS. Regarding to the expression of γH2AX, it predicted a 2.774-fold (95% CI: 1.768–4.352, *P* <  0.001) greater risk of shorter OS and a 2.607-fold (95% CI: 1.671–4.066, *P* <  0.001) greater risk of shorter RFS. The prognostic impact for the OS and RFS of tumor stage, and the expression of Nu-Ox-PTP, Cy-Ox-PTP, and γH2AX were analyzed with Kaplan-Meier survival analysis and are presented in Fig. 2.

**Table 2:** Univariate Cox proportional hazards regression analysis for overall survival and relapse-free survival in gastric carcinoma patients

Characteristics	No.	OS	*P*	RFS	*P*
		HR (95% CI)		HR (95% CI)	
Age, y, ≥ 60 (vs < 60)	117/169	1.254 (0.802–1.963)	0.321	1.280 (0.819–2.001)	0.278
Sex, male (vs female)	128/169	0.956 (0.597–1.529)	0.851	0.978 (0.612–1.563)	0.926
CEA, elevated (vs normal)	31/141	1.802 (1.100–2.952)	0.019	1.783 (1.090–2.918)	0.021
CA19–9, elevated (vs normal)	16/140	2.055 (1.109–3.809)	0.022	1.946 (1.051–3.604)	0.034
TNM stage					
I	34/169	1	< 0.001	1	< 0.001
II	44/169	2.154 (0.932–4.976)	0.073	2.146 (0.929–4.958)	0.074
III	64/169	5.773 (2.694–12.370)	< 0.001	6.070 (2.834–13.001)	< 0.001
IV	27/169	8.753 (3.840–19.953)	< 0.001	8.846 (3.884–20.147)	< 0.001
Venous invasion, presence (vs absence)	30/169	3.360 (2.108–5.357)	< 0.001	3.336 (2.094–5.314)	< 0.001
Tumor invasion, AGC (vs EGC)	133/169	3.510 (1.811–6.803)	< 0.001	3.592 (1.853–6.960)	< 0.001
Histologic grade					
WD	10/117	1	0.104	1	0.118
MD	64/117	2.456 (0.756–7.978)	0.135	2.444 (0.752–7.941)	0.137
PD	43/117	3.325 (1.011–10.936)	0.048	3.248 (0.987–10.683)	0.053
Ox-PTP nucleus, positive (vs negative)	115/169	5.799 (3.084–10.905)	< 0.001	5.954 (3.167–11.192)	< 0.001
Ox-PTP cytoplasm, positive (vs negative)	82/169	1.863 (1.238–2.803)	0.003	1.982 (1.319–2.978)	< 0.001
ɣH2AX, positive (vs negative)	97/169	2.774 (1.768–4.352)	< 0.001	2.607 (1.671–4.066)	< 0.001

Abbreviations: *OS* overall survival, *RFS* relapse-free survival, *HR* hazard ratio, *CEA* carcinoembryonic antigen, *CA19–9* carbohydrate antigen 19–9, *LN* lymph node, *EGC* early gastric cancer, *AGC* advanced gastric cancer

Multivariate analysis was performed with the factors significantly associated with shorter OS and RFS: preoperative serum level of CEA and CA19–9, tumor stage, venous invasion, tumor invasion, and the expression of Nu-Ox-PTP, Cy-Ox-PTP, and γH2AX. Tumor stage (OS; overall *P* = 0.002, RFS; overall *P* = 0.002), venous invasion (OS; *P* <  0.001, RFS; *P* <  0.001), and Nu-Ox-PTP expression (OS; *P* <  0.001, RFS; *P* <  0.001) were independent predictors of shorter OS and RFS. The Nu-Ox-PTP positivity predicted a 9.929-fold (95% CI; 4.116–23.956) greater risk of shorter OS and a 10.358-fold (95% CI; 4.295–24.978) greater risk of shorter RFS (Table 3).

**Table 3:** Multivariate Cox regression analysis for overall survival and relapse-free survival in gastric carcinoma patients

Characteristics	OS	*P*	RFS	*P*
	HR (95% CI)		HR (95% CI)	
TNM stage				
I	1	0.002	1	0.002
II	1.824 (0.656–5.070)	0.250	1.838 (0.661–5.111)	0.244
III	2.487 (0.946–6.541)	0.065	2.729 (1.040–7.159)	0.041
IV	5.066 (1.869–13.736)	0.001	5.208 (1.922–14.113)	0.001
Venous invasion, presence (vs absence)	3.744 (2.109–6.645)	< 0.001	3.752 (2.119–6.644)	< 0.001
Ox-PTP nucleus, positive (vs negative)	9.929 (4.116–23.956)	< 0.001	10.358 (4.295–24.978)	< 0.001

Abbreviations: *OS* overall survival, *RFS* relapse-free survival. Variables considered in multivariate analysis were the pretreatment serum level of CEA and CA19–9, TNM stage, tumor invasion (EGC versus AGC), venous invasion, nuclear expression of Ox-PTP, cytoplasmic expression of Ox-PTP, and ɣH2AX expression

### The combined expression patterns of nuclear ox-PTP and γH2AX predict survival of gastric carcinoma patients

**Fig. 3:**
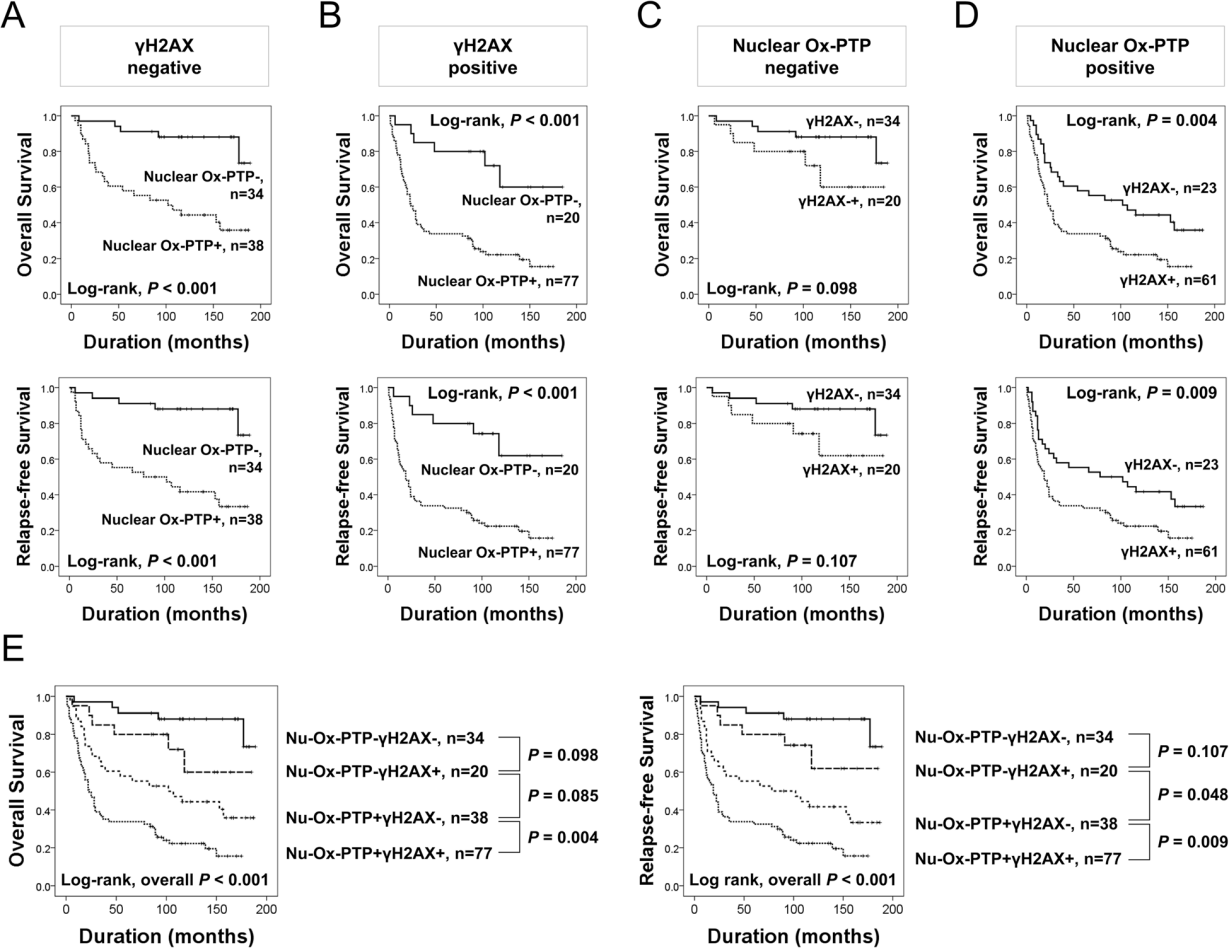
Survival analysis of subgroups of gastric carcinomas according to nuclear Ox-PTP and γH2AX expression. Kaplan-Meier survival analysis for the overall survival and relapse-free survival in γH2AX-negative (**a**), γH2AX-positive (**b**), nuclear Ox-PTP-negative (**c**), and nuclear Ox-PTP-positive (**d**) subgroups. **e** Kaplan-Meier survival analysis in Nu-Ox-PTP^−^γH2AX^−^, Nu-Ox-PTP^−^γH2AX^+^, Nu-Ox-PTP^+^γH2AX^−^, and Nu-Ox-PTP^+^γH2AX^+^ subgroups

**Fig. 4:**
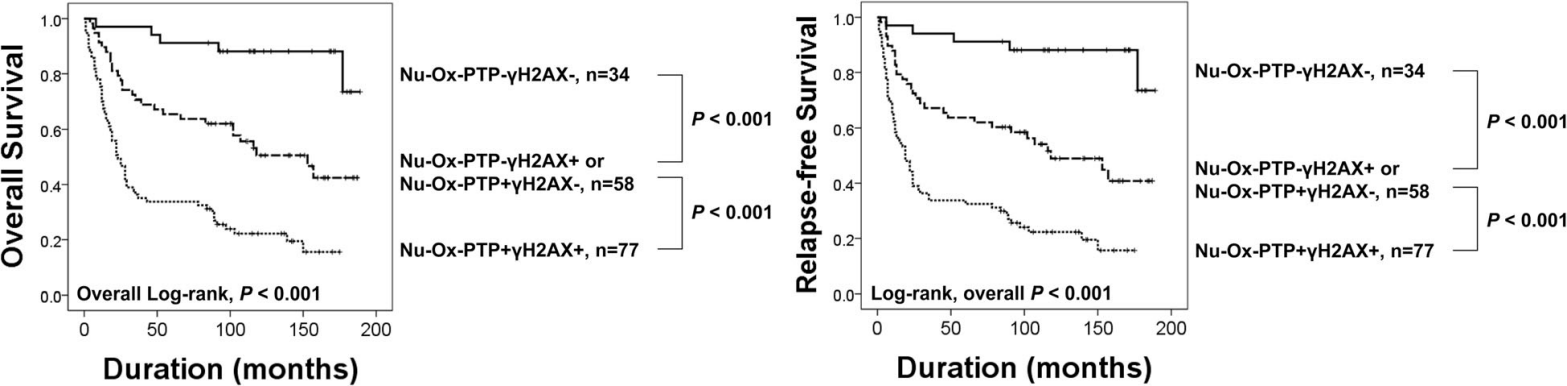
Kaplan-Meier survival analysis in three subgroups according to the combined expression patterns of nuclear Ox-PTP and γH2AX. The subgroups were Nu-Ox-PTP^−^γH2AX^−^, Nu-Ox-PTP^−^γH2AX^+^ or Nu-Ox-PTP^+^γH2AX^−^, and Nu-Ox-PTP^+^γH2AX^+^

In addition to the theoretical relationship and positive correlation between nuclear Ox-PTP and γH2AX expression, both nuclear Ox-PTP and γH2AX expression were independent predictors of shorter OS of gastric carcinoma. Therefore, we further analyzed subgroups of gastric carcinomas according to the nuclear Ox-PTP and γH2AX expression patterns. In both γH2AX-negative and γH2AX-positive subgroups, Nu-Ox-PTP positivity was significantly associated with shorter OS and RFS (Fig. 3a, b). The expression of γH2AX was significantly associated with shorter OS and RFS in the Nu-Ox-PTP-positive subgroup but not in the Nu-Ox-PTP-negative subgroup (Fig. 3c, d). Based on these subgroupings, the gastric carcinomas were grouped as Nu-Ox-PTP^−^γH2AX^−^, Nu-Ox-PTP^−^γH2AX^+^, Nu-Ox-PTP^+^γH2AX^−^, and Nu-Ox-PTP^+^γH2AX^+^. In these subgroups, the combined expression pattern of nuclear Ox-PTP and γH2AX was significantly associated with both OS and RFS in univariate (OS; overall *P* <  0.001, RFS; overall *P* <  0.001) and multivariate analysis (Multivariate analysis Model 1, OS; overall *P* <  0.001, RFS; overall *P* <  0.001) (Table 4). However, despite significance in overall *P* values (OS; overall *P* <  0.001, RFS; overall *P* <  0.001), the difference in survival between each subgroup was not significant, especially between Nu-Ox-PTP^−^γH2AX^−^ and Nu-Ox-PTP^−^γH2AX^+^ (Fig. 3e). Therefore, we re-grouped tumors into three group by combining two groups showing intermediate survival: Nu-Ox-PTP^−^γH2AX^−^, Nu-Ox-PTP^−^γH2AX^+^ or Nu-Ox-PTP^+^γH2AX^−^, and Nu-Ox-PTP^+^γH2AX^+^. With this subgrouping, the prognosis was significantly different in each subgroup (Fig. 4). In univariate analysis, the Nu-Ox-PTP^−^/γH2AX^+^ or Nu-Ox-PTP^+^/γH2AX^−^ subgroup predicted a 4.405-fold (95% CI; 1.703–11.395, *P* = 0.002) greater risk of death, and a 4.609-fold (95% CI; 1.786–11.895, *P* = 0.002) greater risk of relapse or death compared with the ‘Nu-Ox-PTP^−^γH2AX^−^’ subgroup (Table 4). Moreover, the Nu-Ox-PTP^+^γH2AX^+^ subgroup predicted an 11.498-fold (95% CI; 4.572–28.917, *P* <  0.001) greater risk of death, and an 11.494-fold (95% CI; 4.517–28.543, *P* <  0.001) greater risk of relapse or death compared with the Nu-Ox-PTP^−^γH2AX^−^ subgroup (Table 4). In multivariate analysis performed with co-expression pattern of Nu-Ox-PTP and γH2AX, the co-expression pattern of Nu-Ox-PTP and γH2AX was an independent indicator of poor prognosis of OS (overall *P* <  0.001) and RFS (overall *P* <  0.001) (Multivariate analysis Model 2 in Table 4).

**Table 4:** Univariate and multivariate analysis for the overall survival and relapse-free survival according to the combined expression patterns of the nuclear expression of Ox-PTP and ɣH2AX

Characteristics	No.	OS	*P*	RFS	*P*
		HR (95% CI)		HR (95% CI)	
Univariate analysis					
Nu-Ox-PTP/ɣH2AX					
−/−	34/169	1	< 0.001	1	< 0.001
−/+	20/169	2.503 (0.762–8.224)	0.131	2.455 (0.747–8.067)	0.139
+/−	38/169	5.471 (2.079–14.402)	< 0.001	5.876 (2.240–15.412)	< 0.001
+/+	77/169	11.420 (4.545–28.697)	< 0.001	11.273 (4.489–28.311)	< 0.001
Nu-Ox-PTP/ɣH2AX					
−/−	34/169	1	< 0.001	1	< 0.001
−/+ or +/−	58/169	4.405 (1.703–11.395)	0.002	4.609 (1.786–11.895)	0.002
+/+	77/169	11.498 (4.572–28.917)	< 0.001	11.354 (4.517–28.543)	< 0.001
Multivariate analysis Model 1^a^					
TNM stage,					
I		1	0.003	1	0.003
II		1.719 (0.617–4.790)	0.300	1.746 (0.627–4.865)	0.287
III		2.417 (0.917–6.372)	0.074	2.647 (1.007–6.956)	0.048
IV		4.725 (1.739–12.839)	0.002	4.858 (1.785–13.219)	0.002
Venous invasion, presence (vs absence)		3.674 (2.056–6.564)	< 0.001	3.737 (2.098–6.658)	< 0.001
Nu-Ox-PTP/ɣH2AX					
−/−		1	< 0.001	1	< 0.001
−/+		2.822 (0.523–15.882)	0.224	2.817 (0.511–15.527)	0.234
+/−		13.147 (2.992–57.772)	< 0.001	14.430 (3.294–63.208)	< 0.001
+/+		20.309 (4.801–85.912)	< 0.001	20.434 (4.831–86.435)	< 0.001
Multivariate analysis Model 2^a^					
TNM stage					
I		1	0.002	1	0.002
II		1.668 (0.597–4.663)	0.329	1.682 (0.602–4.701)	0.322
III		2.909 (1.108–7.643)	0.030	3.171 (1.210–8.310)	0.019
IV		5.009 (1.848–13.579)	0.002	5.058 (1.864–13.722)	0.001
Venous invasion, presence (vs absence)		2.849 (1.649–4.920)	< 0.001	2.837 (1.646–4.890)	< 0.001
Nu-Ox-PTP/ɣH2AX					
−/−		1	< 0.001	1	< 0.001
−/+ or +/−		7.882 (1.847–33.640)	0.005	8.297 (1.948–35.334)	0.004
+/+		18.120 (4.311–76.156)	< 0.001	17.970 (4.279–75.470)	< 0.001

Abbreviations: *OS* overall survival, *RFS* relapse-free survival, *Nu-Ox-PTP* nuclear expression of oxidized-PTP. ^a^Variables considered in multivariate analysis Model 1 and 2 were the pretreatment serum level of CEA and CA19–9, TNM stage, tumor invasion (EGC versus AGC), venous invasion, and the combined expression patterns of nuclear expression of Ox-PTP and ɣH2AX

## Discussion

In this study, we have shown that the expression of Ox-PTP and γH2AX are predictive for the prognosis of gastric carcinoma patients. Especially, nuclear expression of Ox-PTP was an independent indicator of poor prognosis for the OS and RFS of gastric carcinoma patients, and γH2AX positivity also predicted shorter OS. Moreover, the co-expression pattern of Nu-Ox-PTP and γH2AX was also an independent indicator of poor prognosis of OS and RFS.

The prognostic impact of Ox-PTP expression in gastric carcinomas was associated with its biologic role and activity in cancer cells. PTPs regulate the phosphorylation status of tyrosine residues in proteins involved in cell signaling pathways and, in principle, act as tyrosine kinase antagonists [34, 35]. In addition, PTPs are involved in oxidative stress-mediated regulation and are inactivated through nucleophilic attack [1, 5]. Aberrant activation of human growth factor signals EGFR, PDGFR and IGF-1R stimulate ROS production, which inhibits PTPs and stimulates phosphorylation of receptor tyrosine kinases [36, 37]. In this aspect, the poor prognosis of Ox-PTP-expressing gastric carcinomas might be related with irreversible inactivation of PTP by hyper-oxidation [37, 38] or higher levels of oncogenic PTP in the cancer itself [28, 39–41].

With respect to inactivation of PTP by hyper-oxidation in ROS-mediated carcinogenesis, PTPN12 has been suggested as a tumor suppressor in triple-negative breast cancer [42]. PTPN12 suppressed the transformation and proliferation of epithelial cells by inhibiting receptor tyrosine kinases including HER2/EGFR-MAPK signaling. In addition, depletion of PTPN12 led to hyper-phosphorylation of EGFR and HER2 phosphorylation and subsequent activation of the RAS/MAPK signaling in mammary epithelial cells [42]. However, the expression pattern of PTP was different according to the type of PTP. In gastric tissue, the expression of PTPN4, PTPRA, and PTPRS were increased in cancer tissue compared with normal tissue [43]. In contrast, the expression of PTPRC and PTPRG were decreased in cancer tissue compared with normal tissue [43]. Moreover, the roles of PTPs in tumorigenesis are controversially reported according to the type of PTPs and some PTPs have been reported to have oncogenic potential. Overexpression of PRL-2 phosphatase promoted spontaneous tumorigenesis in breast [44], and knock-down of PTP1B with siRNA inhibited proliferation of MCF-7 breast cancer cells [45]. In addition, the expression of PRL-3 mRNA was higher in colorectal cancers compared with normal colonic mucosa [39], which was higher in metastatic lesions compared with primary colorectal cancers [40]. Moreover, immunohistochemical expression of PRL-3 predicted shorter survival of breast cancer patients [41]. In gastric cancers, higher expression of PRL-3 was associated with peritoneal metastasis and shorter survival of patients [28]. In this respect, poor prognosis of Ox-PTP-expressing cancers might be related with higher levels of PTP expression in established cancers. Therefore, further study is needed to clearly define the effects of oxidation of PTP in the development and progression of human malignant tumors.

**Fig. 5:**
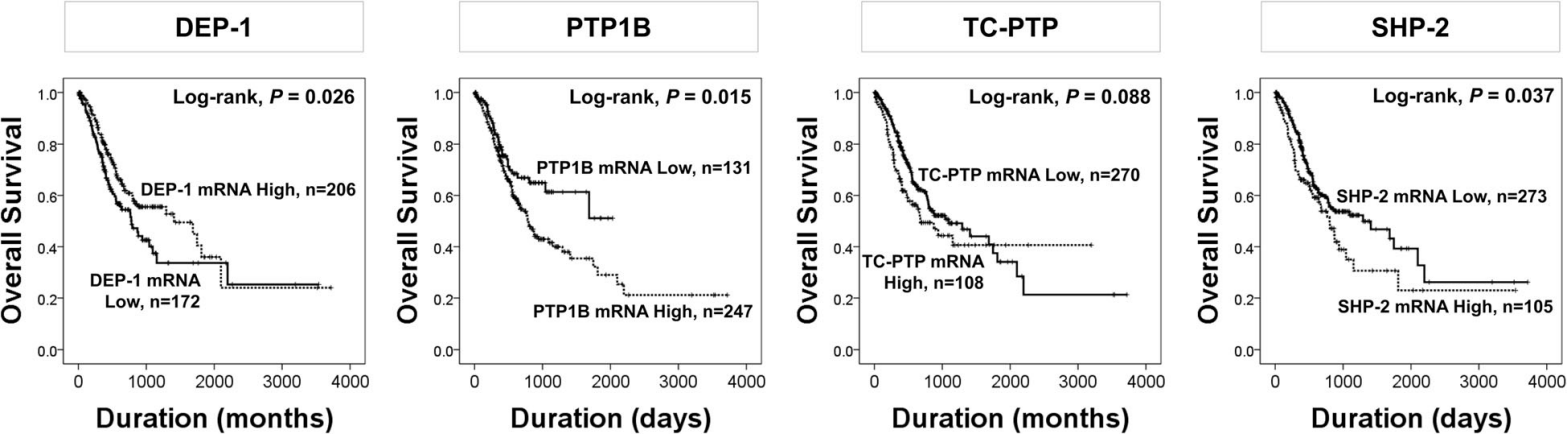
Kaplan-Meier survival analysis according to the mRNA expression of DEP-1, PTP1B, TC-PTP, and SHP-2 in gastric carcinomas. The data for the mRNA levels of DEP-1, PTP1B, TC-PTP, and SHP-2, and survival data of gastric carcinoma patients were obtained from the OncoLnc database (http://www.oncolnc.org. Accessed 2 March 2018)

Concerning the localization of PTP, it could be in the cytoplasm, or in both the cytoplasm and nucleus according to the type of PTP [2]. Therefore, the expression of Ox-PTP could be observed in both localizations, and our results showed cytoplasmic and nuclear expression of Ox-PTP in gastric cancer cells. In addition, both nuclear and cytoplasmic expression of Ox-PTP were associated with shorter survival of gastric carcinoma patients. However, Nu-Ox-PTP positivity was more predictive for the estimation of survival of gastric carcinomas compared with Cy-Ox-PTP positivity. Based on the information for the antibody used for detection of Ox-PTP, the anti-Ox-PTP antibody can detect oxidized DEP-1, PTP1B, TC-PTP, and SHP-2. Among these four PTPs, DEP-1 and PTP1B was expected to be expressed in the cytoplasm, and TC-PTP in the nuclei, and SHP-2 in both nuclei and cytoplasm of cells based on public data base from “The Human Protein Atlas” (THPA; https://www.proteinatlas.org, accession date; 2 March 2018). Therefore, when considering the prognostic significance of Nu-Ox-PTP, hyper-oxidation of nuclear-type PTP such as TC-PTP and SHP-2 might be important in the progression of gastric carcinomas. In this context, based upon the assumption that hyper-oxidation-mediated change on the activity of nuclear PTPs, the expression of PTPs might also be associated with the progression of human malignant tumors. However, studies on the prognostic impact of PTP expression in human cancers have been limited, and the results are controversial. The expression of TC-PTP mRNA was associated with favorable prognosis in ovarian cancers (THPA database, Log-rank, *P* <  0.001), but predicted shorter OS in clear cell renal cell carcinoma (THPA database, Log-rank, *P* <  0.001) based on THPA database using the TCGA database. In addition, higher expression of DEP-1 mRNA was associated with favorable prognosis of clear cell renal cell carcinoma patients (THPA database, Log-rank, *P* <  0.001, accession date; 2 March 2018). In addition, when we evaluated the prognostic significance of mRNA expression of DEP-1, PTP1B, TC-PTP, and SHP-2 in gastric carcinomas by using OncoLnc database (http://www.oncolnc.org), higher expression of DEP-1 mRNA expression was associated with longer survival (Log-rank, *P* = 0.026) (Fig. 5). In contrast, higher mRNA expression of PTP1B (Log-rank, *P* = 0.015) and SHP-2 (Log-rank, *P* = 0.037) were associated with shorter survival of gastric carcinomas; however, there was no correlation survival and TC-PTP expression (Log-rank, *P* = 0.088) (Fig. 5). The difference between our results and those of other databases might be related with the post-translation modification of PTP proteins by hyper-oxidation, which have an important role in addition to the gene expression level itself. In addition, regarding the prognostic significance of Nu-Ox-PTP expression compared with Cy-Ox-PTP expression, these expression levels may correlated with the composition of PTPs localized in the cytoplasm and/or nuclei, and the translocation of PTPs between the cytoplasm and nuclei according to the type of PTPs. However, our results are limited by the fact that the antibody used to detect Ox-PTP does not specify the type of PTP according to its subcellular localization. However, despite this limitation, our results suggest that oxidative stresses, as posttranslational modifiers, might have an important role in gastric carcinogenesis and potentially to be therapeutic target. However, further study is needed to clarify the precise role of PTPs and their oxidation in human cancers.

Another finding of this study is that the expression of Ox-PTP and γH2AX were significantly associated in gastric carcinoma tissue. Additionally, western blotting indicated increased expression of Ox-PTP and γH2AX with H_2_O_2_ treatment (Fig. 1a). This finding might be due to the fact that both oxidation of PTP and expression of γH2AX as DDR might be common consequences of oxidative stress-mediated damage of cells [46]. In addition, the expression of both Ox-PTP and γH2AX were significantly associated with the progression of gastric carcinoma such as higher tumor stages, lymph node metastases, and shorter survival. Consistently, higher expression of γH2AX was associated with higher stage and lymph node metastasis of gastric cancers [47]. Moreover, γH2AX expression predicted shorter survival in various human malignant tumors, such as breast carcinomas [22, 23], soft-tissue sarcomas [21], endometrial carcinomas [20], and non-small cell lung cancers [48]. The poor prognosis of γH2AX-expressing cancers might be related with the fact that γH2AX confer resistance to cancer cells against conventional genotoxic anti-cancer therapies by repairing DNA damage. In this context, the combined expression patterns of DDR molecules expressed in single- and double-strand breaks of DNA such as PARP1, γH2AX, and BRCA1/2 were indicators of poor prognosis of breast carcinomas and soft-tissue sarcomas [21, 23]. Similarly, the co-expression pattern of Ox-PTP and γH2AX was very predictive for the estimation of prognosis of gastric carcinoma patients. Gastric carcinoma patients possessing tumors positive for both Ox-PTP and γH2AX had the shortest survival. The five- and ten-year OS rates of the Nu-Ox-PTP^−^γH2AX^−^ subgroup was only 34% and 22%, respectively. In contrast, the five- and ten-year OS rates of the Nu-Ox-PTP^−^γH2AX^−^ subgroup was 91% and 88%, respectively. The OS rate of the Nu-Ox-PTP^−^/γH2AX^+^ or Nu-Ox-PTP^+^/γH2AX^−^ subgroup was intermediate (five-year OS rate; 66%, ten-year OS rate; 50%). Therefore, as a common consequence of oxidative stresses, our results suggest that the combined expression patterns of Ox-PTP and γH2AX might be useful in the prediction of prognosis of cancer patients.

## Conclusions

In summary, our results suggest that the accumulation of irreversibly hyper-oxidized PTP and γH2AX might be involved in the progression of gastric carcinoma. Moreover, based on survival analysis, our results suggest that the individual and co-expression patterns of Ox-PTP and γH2AX might be useful as a prognostic marker for gastric carcinoma patients. In addition, although this study had the limitation that the staining for Ox-PTP could not specify specific PTPs involved in the progression of gastric carcinoma, our results suggest that regulation of PTPs also could be potential target for the treatment of gastric carcinoma. Therefore, further study is needed to verify the specific types of PTP which are primarily involved in gastric carcinomas in order to refine a therapeutic approach. Moreover, despite the limitation that the expression of Ox-PTP was evaluated by semi-quantitative immunohistochemical staining which does not delineate the type of PTPs oxidized in gastric cancers, to our knowledge this is the first study to evaluate oxidized PTPs in human cancer tissue and to elucidate their potential as a prognostic marker of gastric carcinomas.

## Abbreviations

95% CI: 95% confidence interval
CA 19–9: Cancer antigen 19–9
CEA: Carcinoembryonic antigen
Cy-Ox-PTP: Cytoplasmic expression of oxidized-protein tyrosine phosphatase
DDR: DNA damage response
Nu-Ox-PTP: Nuclear expression of oxidized-protein tyrosine phosphatase
OS: Overall survival
Ox-PTP: Oxidized-protein tyrosine phosphatase
PTP: Protein tyrosine phosphatase
RFS: Relapse-free survival
THPA: The Human Protein Atlas
γH2AX: Phosphorylated-H2AX
